# Cure of Femoral Aneurism, Treated First by Compression, and Subsequently by Ligation of the External Iliac

**Published:** 1875-04

**Authors:** C. C. F. Gay

**Affiliations:** Surgeon Buffalo Generai Hospital


### Case of Femoral Aneurism treated first by Compression and subsequently by Ligation of the External Iliac Artery.

By C. C. F. Gay, M. D., Surgeon Buffalo General Hospital. Reported by Bernard Bartow, M. D., Resident Physician.

Richard H., æt. 36, applied to be admitted to the Buffalo General Hospital March 24th, 1874, having a pulsating tumour on the inner side of the left thigh, four inches below Poupart's ligament. It was about double the size of an adult fist, painful, burning in character, with shooting pains extending down the limb. The pulsation could be discerned by the eye, and when the hand was applied, showed its expansive nature, peculiar to aneurism, seeming uniform throughout, exeept at one point upon the inner and lower aspect of the tumour about opposite the mouth of the sac—where its fluid contents could be felt—the pulsation was strongest. A distinct thrill could be felt at the upper portion of the tumour, and extending upwards two inches, in a line corresponding with the course of the femoral artery. The ear or stethoscope placed upon the tumour showed the presenee of a "bruit," most distinctly heard at the upper margin, and varying in intensity with the position of the limb; being loudest when the whole limb was raised to an angle of forty-five degrees. The diagnosis was plainly aneurism of the superficial femoral artery, and the mouth of the sac was not more than one inch below the origin of the deep femoral branch. The patient had been engaged making steam boilers, and was employed to hold an iron bar—the "miser"—against which the boiler bolts were flattened by hammering. To hold the bar more securely, he had frequently rested one end of it against his thigh, where the aneurism now is, which had received the shock of the hammer. It was evidently of traumatic origin.

His attention was directed to his disease in October of last year, by experiencing sudden lancinating pain in the part; two or three weeks after, he noticed a small tumour, the size of a hazel-nut, in the same place. It was not painful at that time, and gave him no uneasiness until he noticed that it was increasing in size, and becoming painful proportionately. He continued to work notwithstanding the continued enlargement and increase of pain, until entering the hospital—a period of five months.

The condition of the patient was precarious, and not such as to bear a severe operation, being exhausted by pain and sleeplessness.

Operative procedures were deferred until his strength could be recruited, which took about ten days to accomplish. The tumour was meanwhile securely held by imbricated strips of adhesive plaster ; absolute rest being enjoined upon the patient.

Ligature of the common femoral, or of the external iliac arteries, were the only operative sources from which to choose ; the former was discarded from the liability to secondary hemorrhage.

Before taking this grave step it was thought expedient to employ the treatment by compression, it having been shown that where occlusion of the sac does not follow its use, it causes but little additional disturbance, is without danger, and enhances the probability of recovery from ligation by enlarging the collateral circulation, before the old circulation is shut off, lessening thereby the danger of gangrene.

Digital compression was, accordingly, begun April 4th, and was continued for thirty-one of the following forty-eight hours : seventeen hours being consumed by the patient in sleep, at intervals of from one to six hours. The pain was subdued by hypodermic injections of morphia, gr. $\frac{1}{4}$ to $\frac{1}{3}$, every three or four hours.

The degree of pressure was not such as to completely prevent circulation through the sac, a small amount being allowed to enter, which was regulated by the force of the pulsation and "bruit." These could be made to cease by making the requisite pressure.

The first effect was to render the whole limb livid ; it became perceptibly cooler than the sound one. Patient complained of coldness, numbness, and tingling of the limb, with shooting pains extending from the point of pressure down to the ankle—due probably to pressure upon some branches of the anterior crural nerve. At the end of eight hours the colour and warmth returned ; the pulsation of any of the arteries below the tumour could not be felt, whereas they were perceptible before the pressure was begun ; the tumour had increased in firmness, and the removal of the pressure showed considerable diminution of the force of the pulsation and "bruit."

The following morning, sixteen hours from commencement, the pulsation had diminished to an extent that the patient was free from pain when not under the influence of pressure, which had not been so at any time since his admission notwithstanding the free use of anodynes, nor did he complain of the pulsation giving rise to pain at any time during his subsequent treatment. At the expiration of forty-eight hours the whole limb had become quite œdematous, especially around and below the lower part of the tumour, giving it the appearance of having become diffused.

There was constantly present a tingling and burning sensation, which began at the toes and extended to the groin, and would be aggravated when the foot was touched in any of the manipulations. The tumour had become quite dense ; the pulsation and bruit were reduced nearly to one-half their former intensity, and in some places could not be felt or heard where they previously existed. At this point digital compression was abandoned from scarcity of assistants; instrumental compression being substituted. The apparatus used was constructed upon the principle of a lever of the first kind; the fulcrum resting upon the artery and forming the compressor.

With the pad in position and the power applied, no movement on the part of the patient could displace it; remaining fixed until the power was relaxed. The irritation of the pad was less than that caused by the frequent changing of the thumbs of the assistants while making digital compression—causing after a time the excoriation of the skin. This was continued for period's of six hours, alternating with six hours' intermission for seven days, causing the pulsation and bruit to become reduced to one-fourth of their greatest intensity. The circulation as before was not completely shut off from the sac.

The area of surface to which pressure could de applied was one inch in breadth by two and one-half inches long, which was insufficient to allow of a requisite amount of rest to the parts in the intervals. The space had become very irritable and inflamed at the end of this time, and it was necessary to suspend pressure for two days, the effect of which was to lose nearly one-half of the advantage gained, which appeared very promising at the time of the discontinuance.

Pressure was resumed at the same point, but could not be made to the same degree from tenderness of the integument, and was only sufficient to prevent a complete relapse.

Pressure was now removed to the external iliac artery where it crosses the pectineal eminence. It required, however, a greater amount of power to produce the same effect, increasing thereby the danger of sloughing.

The principle kept in view thus far had been to retard the current through the sac, that the deposition of laminæ of coagulum might take place gradually. This failing to occlude the sac, after having been fairly tried, and it now being evident that pressure could be maintained but for a short time, it was decided io try the effect of *stopping* the circulation through the aneurism. The increased pressure caused severe pain, and required large doses of morphia to enable the patient to bear it, which he did for fifteen consecutive hours.

The œdema of the limb increased, and upon the removal of pressure the bruit could be heard faintly at the upper part of the tumour, nearly over the mouth of the sac; but pulsation was not apparent, the tumour being hard and elastic. At the expiration of the following twenty-four hours the pulsation returned, though pressure had been continued at intervals sixteen hours of this time. Forty-eight hours from the time when pulsation ceased, pressure was abandoned from the irritability of the compressed surfaces. This is to be regretted, as more progress had been made during that time than in the eleven previous days.

As a last resort before operating, flexion of the thigh upon the abdomenn was tried. This controlled the circulation completely, but it also had to be abandoned after six hours, the patient suffering more during this period than from any of the previous methods employed. These forms of treatment had been continued for twelve days, and during more than half of this time the arteries were undergoing compression.

The patient complaind of no pain in the tumour or limb at this time, and his general condition was even improved. The measurement around the thigh over the site of the tumour was twenty-two inches, no diminution having followed the treatment. The amount of effusion around the lower part of the tumour increased out of all proportion to that in the remainder of the limb, and it was considered certain by some who examined it that the aneurism had become diffused. Nothing in the appearance of the patient occurred to indicate so grave an accident other than the enlargement, which subsequently disappeared, showing the circumscribed character of the tumour.

*April* 14, 1874. Dr. Gay, in the presence of a large number of medical gentleman, ligated the external iliac artery. The "operation from below" (Cooper's) was the one chosen. When the peritoneum was reached it very much resembled the transversalis fascia, being thickened and opaline in colour, due to the pressure made upon the external iliac artery, giving rise to circumscribed inflammation at that point; owing to this abnormal appearance the peritoneum was wounded. The pulsation and bruit ceased immediately after the ligature was tightened.

The wound was closed by a few interrupted sutures, none of which, however, included the peritoneum where it was wounded. Patient rallied well from the operatión ; felt a pricking or tingling sensation in his instep and ankle, extending to the knee ; the limb did not change its colour, and only half a degree difference in temperature was shown by the thermometer in the popliteal spaces ; no increase of the œdema followed ; warmth was applied to the limb by means of bottles of hot water, which was grateful to the patient ; the whole surface of the abdomen was covered with a poultice of hops ; opium and stimulants being freely administered. No tympanitis or signs of general peritonitis followed ; around the margins of the wound it was very tender, showing the existence of circumscribed inflammation. Primary union was obtained in the upper part of the wound ; sutures were removed the fifth day ; lower portion of wound gaped ; the edges were approximated by flexing the thighs and raising the shoulders, which also allowed a more free escape of pus.

Two days after operation, the measurement around thigh, over the tumour, was eighteen inches, being a reduction of four inches in circumference. No pulsation could be distinguished in any of the arteries of the limb. The pricking and burning sensations continued throughout the treatment, being at times actually painful. The ligature came away on the fourteenth day, after which the wound rapidly healed.

Five weeks after operation, patient was able to be out of doors, but was obliged to use crutches on account of a slough upon the the back of the heel, the size of a silver dollar, and another upon the great toe. The former penetrated to the bone, and it was, on this account, four months before he could walk without the assistance of crutches.

No signs of suppuration of the contents of the sac followed the ligation ; the sac being of a firm, elastic consistence, and free from pain. The measurement of the thigh, at the site of tumour, further diminished to 17½ inches in circumference, being the least it reached ; the thigh after this became more fleshy.

The tumour itself continued to be absorbed, so that its circumscribed form could be more distinctly defined. When patient left hospital, October 1, 1874, it was of the dimensions and shape of the umbrella portion of a moderate sized mushroom. It caused but slight enlargement at that part of thigh, and would not be noticed in a casual observation.

The cicatrix of the upper part of the wound, at which point the peritoneum was divided, appears weak. Patient wore a compress and bandage while in hospital, but was advised on going out to wear a truss, as a precautionary measure to prevent hernial protrusion. Some authors refer this sequel to not including the peritoneum in the sutures, which, as before mentioned, was not done in this case. Hernia followed from a similar cause, in an otherwise successful case, where the external iliac artery was tied by Mr. Kirby.*

The results of this case show the beneficial influence of pressure, and furnish evidence of its value, and sufficient reason why it should be employed in all cases of aneurism when practicable. Had this aneurism been of smaller size compression would probably have superseded the necessity of ligating the artery ; or, with an aneurism of the same size, and a greater area upon which to make a pressure, there is reason to believe that compression alone would be attended with success.

It corroborates the views of Holmes in regard to the influence of compression in more speedily promoting the new circulation.

The apparatus used in this case answers the purpose of the various and expensive contrivances used, and is within reach of any one of moderate mechanical ability.—*American Journal Medical Sciences, January*, 1875.

---

* Manual of Operations of Surgery, by Joseph Bell, F. R. C. S.

